# Asymptomatic Host Plant Infection by the Widespread Pathogen *Botrytis cinerea* Alters the Life Histories, Behaviors, and Interactions of an Aphid and Its Natural Enemies

**DOI:** 10.3390/insects9030080

**Published:** 2018-07-06

**Authors:** Norhayati Ngah, Rebecca L. Thomas, Michael W. Shaw, Mark D. E. Fellowes

**Affiliations:** 1 People and Wildlife Research Group, School of Biological Sciences, University of Reading, Whiteknights, Reading, Berkshire RG6 6AJ, UK; 2 Fakulti Biosumber dan Industri Makanan, Universiti Sultan Zainal Abidin, Besut Terengganu 22200, Malaysia; norhayatingah@unisza.edu.my; 3 School of Biological Sciences, Royal Holloway University of London, Egham, Surrey TW20 0EX, UK; rebecca.thomas@rhul.ac.uk; 4 School of Agriculture, Policy and Development, University of Reading, Whiteknights, Reading, Berkshire RG6 6AR, UK; m.w.shaw@reading.ac.uk

**Keywords:** aphid, parasitoid, predator, *Myzus persicae*, *Aphidius colemani*, *Adalia bipunctata*, *Lactuca sativa*, lettuce, biological control

## Abstract

Plant pathogens can profoundly affect host plant quality as perceived by their insect herbivores, with potentially far-reaching implications for the ecology and structure of insect communities. Changes in host plants may have direct effects on the life-histories of their insect herbivores, which can then influence their value as prey to their natural enemies. While there have been many studies that have explored the effects of infection when plants show symptoms of disease, little is understood about how unexpressed infection may affect interactions at higher trophic levels. We examined how systemic, asymptomatic, and seed-borne infection by the ubiquitous plant pathogen *Botrytis cinerea*, infecting two varieties of the lettuce *Lactuca sativa*, affected aphids (the green peach aphid, *Myzus persicae*) and two widely used biocontrol agents (the parasitoid *Aphidius colemani* and the ladybird predator *Adalia bipunctata*). Lettuce varieties differed in host plant quality. Asymptomatic infection reduced chlorophyll content and dry weight of host plants, irrespective of plant variety. Aphids reared on asymptomatic plants were smaller, had reduced off-plant survival time and were less fecund than aphids reared on uninfected plants. Parasitoids showed reduced attack rates on asymptomatically infected plants, and wasps emerging from hosts reared on such plants were smaller and showed reduced starvation resistance. When given a choice in an olfactometer, aphids preferentially chose uninfected plants of one variety (Tom Thumb) but showed no preference with the second (Little Gem) variety. Parasitoids preferentially chose aphids on uninfected plants, irrespective of host plant variety, but ladybirds did not show any such preference. These results suggest that the reduced quality of plants asymptomatically infected by *Botrytis cinerea* negatively affects the life history of aphids and their parasitoids, and alters the behaviors of aphids and parasitoids, but not of ladybirds. Fungal pathogens are ubiquitous in nature, and this work shows that even when host plants are yet to show symptoms, pathogens can affect interactions between insect herbivores and their natural enemies. This is likely to have important implications for the success of biological control programs.

## 1. Introduction

Plant pathogens are ubiquitous in nature, affecting the growth and development of many plant species, and reducing the quality of the plant as experienced by herbivores [1]. They are of enormous economic importance. Besides direct crop loss, they cause indirect losses through the cost of prevention and treatment [2]. Infection by plant pathogens frequently affects the respiration and transpiration capabilities of host plants, resulting in decreased rates of photosynthesis, which in turn alters rates of nutrient translocation, causing a net influx of nutrients into infected tissues [3]. Many plant species also react to pathogen infection by triggering a change in the rate of hormone synthesis or degradation [4]; these changes in turn alter the production of secondary defenses and alter the host plant’s normal resistance pathways [5,6,7]. 

As plant pathogens and herbivorous insects may share the same host plant, changes in plant traits caused by infection can act as a feeding deterrent to herbivorous insects, and can also alter their physiology and development, resulting in reduced growth rates, reduced adult size, and increased mortality rates [8,9]. Most notably, chewing insects and necrotrophic pathogens (e.g., *Botyrtis*) can induce the jasmonic acid (JA) dependent defense pathway, while sap-sucking insects, and biotrophic pathogens, induce the salicylic acid (SA) dependent defense pathway [10]. As these pathways crosstalk [11], attack by one pathogen or herbivore can induce defenses that affect another, changing perceived plant quality [12]. Pathogen infection may also influence insect behavior. For example, pathogen infection interferes with plant volatile emission profiles (VOCs; [13,14]) and visual cues used by insects if infection alters plant morphology [15]. Both of these cues play an important role in mediating ecological interactions among plants and insects [16,17,18,19], particularly in terms of host plant location and choice.

However, plant pathogen infection can also have a positive effect on the fitness, performance and host plant preference of insect herbivores [20,21,22,23]. Herbivorous insects may benefit from pathogen infection when the presence of the pathogen increases nutrient levels (e.g., by digesting the complex sugars in infected leaves) or when the pathogenic fungi changes the plant defense mechanisms in a way that makes it more susceptible to the insect herbivore [1]. These effects may also have consequences at higher trophic levels, with the predators and parasitoids of insect herbivores are in turn affected by consequent changes in the quality of their hosts [24,25]. For example, pathogen infection can cause a change in the composition of plant volatiles [26], which in turn alters their attraction to parasitoids [27]. If parasitoids attack hosts on pathogen-infected plants, then they may alter their sex allocation behavior to reflect perceived differences in host quality, as female parasitoids can choose to place male eggs in relatively poor quality hosts [28,29]. Furthermore, when host quality varies, female parasitoids are expected to preferentially oviposit in high-quality hosts [29,30] and parasitoids emerging from lower quality hosts can experience higher levels of mortality and grow into smaller adults [31]. Similar patterns of behavior may be seen with insect predators, where prey quality may influence behavior [29]. Therefore, plant pathogen infections can have effects that extend well beyond the direct effects that they exert on the physiology and life-history of their host plants. 

However, plant pathogen infections do not always result in visually obvious negative effects on the plant, such as defoliation or wilting of the leaves, which could affect the visual preferences of insects [32]. Pathogen infection in which a live pathogen is present in a host but does not cause gross damage to the host is referred to as a latent infection. Latency can occur at any stage of the crop life cycle and at any stage of pathogen growth [33,34]. Latent infection may take the form of quiescence, surviving but not growing until appropriate environmental triggers are perceived [35]. Alternatively, the pathogen may grow inside the host plant but still cause no or very slight symptoms. A number of studies have suggested that a range of plant species such as wheat [36], grapes [37], basil [38], and woody plants [39,40] harbor such hidden infections by plant pathogens. We have little understanding of the consequences—whether positive or negative—of such hidden infections for insect herbivores and in turn their predators and parasitoids. This is a fundamental question of considerable interest, given the ubiquity of plant pathogens. 

The common and widespread generalist plant pathogen *Botrytis cinerea* Persoon: Fries *s. lato* has been the focus of many epidemiological and biocontrol studies (e.g., [41,42,43]), and causes extensive damage to a wide range of economically important crops worldwide [44]. Known as ‘grey mold fungus’, this airborne fungus attacks over 200 plant species [45]. Infection by this pathogen will either reduce or eliminate the marketability of the harvested product [44]. *Botrytis cinerea* multiplies through conidia that directly infect the host plant, typically resulting in spreading necrotrophic lesions [44]. Once it has penetrated into the plant system, *B. cinerea* secretes a range of nonspecific chemical compounds, including oxalic acid [46], the fungal toxin botrydial [47], and hydrogen peroxide [48]. These compounds contribute to host-plant cell death and promote the growth of macerated lesions [49]. In turn, the plants then activate resistance mechanisms to combat this pathogen attack [50]. One of the plants first defenses is to activate the hypersensitive response (HR), generating the oxidative burst that can trigger hypersensitive cell death [51]. 

*Botrytis cinerea* also has been seen to remain quiescent in strawberry leaf epidermal cells [52], grape flowers [53], and quiescent infection, in which a few dead cells harbor localized but live *B. cinerea*, limited by host defenses in the surrounding cells is probably a common cause of post-harvest infection in many fruit, including strawberries and raspberries [54]. In some cases, *B. cinerea* can grow systemically, extending as the plant grows, without the plant showing symptoms of infection [55,56]. This plant pathogen is often present in what are otherwise visually healthy lettuce plants [56,57] as an asymptomatic, endophytic infection, which may also arise from seed [58]. As the host plant grows, infection spreads into roots, stem, and leaves [56]. This form of infection is known in multiple *Botrytis* species and in hosts including hybrid primula plants [55], *Pelargonium* sp. leaves [59], wild *Primula*, and *Taraxacum vulgare* agg. (dandelion) [54,60], and daylilies [61]. The likelihood of quiescent infection by *B. cinerea* varies among plant species. 

Asymptomatic infection by *B. cinerea* may still alter host plant physiology. This may then have a consequential effect on organisms at a higher trophic level. However, despite the potential ubiquity of hidden infection, little is known of the consequences for insect herbivores and their natural enemies [62]. We addressed this in a laboratory study, using two varieties of lettuce, an economically valuable crop plant (production in the UK alone was valued at >£150 million in 2016; [63]) as our host plants. We asked if asymptomatic infection by *B. cinerea* (i) alters plant traits; (ii) influences the size and life history traits of an insect herbivore (the green peach aphid, *Myzus persicae*) and its parasitoid (*Aphidius colemani*); (iii) affects host/prey choice behavior of the aphid, its parasitoid and a predator (the two-spot ladybird beetle, *Adalia bipunctata*) and (iv) alters the expression of aphid escape behavior when exposed to predator attack.

## 2. Materials and Methods 

### 2.1. Study System

All experiments and plant and insect rearing were carried out in a constant environment (CE) room at 18–20 °C, relative humidity 80 ± 5% and L16:D8 photoperiod. Seeds of two commercial varieties of lettuce *Lactuca sativa* L. (Little Gem and Tom Thumb; Thompson and Morgan, Suffolk, UK; harvest year 2013) were used and grown in 15 cm diameter pots with traditional potting compost (Vitax Grower, Leicester, UK). To ensure that plants used in experiments were otherwise identical, plants were grown from single source seed to produce plants for use in all later experiments. Plants grown for ‘infected seeds’ and ‘uninfected seeds’ were grown separately, to reduce infection rates of our control (uninfected) plants. Infected plants were grown from systemically infected seed collected from plants which had been previously inoculated with *B. cinerea* strain B05.10 at the flowering stage (following [56]), while uninfected plants were grown from uninfected seeds collected from uninfected plants. Six-week-old plants (19 on the BBCH scale), which were free from any symptoms of disease, were used in this study. Sixty replicates were set up per treatment, as it proved impossible to both guarantee infection in the treated plants and lack of infection in the control plants. A week before each experiment started, plant infection status of the plants was checked using *Botrytis* Selective Media Agar (BSM). Thirty infected/uninfected plants were then selected randomly from the tested plants for use in trials. It should be noted that some plants did show symptoms of infection, so final replicate numbers for some insect trials were lower than 30.

*Insect life history traits.* Both species of insects were reared in rectangular clear plastic cages (20 cm × 20 cm × 15 cm) fitted with the cotton mesh windows. A monoclonal culture of the green peach aphid *Myzus persicae* Sulzer (Hemiptera: Aphididae), which had been locally collected from cabbage plants and had been in culture for several years prior to this experiment, was used in this and the following experiments. Aphids were reared on both varieties of uninfected and infected lettuce plants for five generations before the experiment, thus avoiding any confounding maternal effects [64]. All aphids used in the experiment were alates. Parasitoids *Aphidius colemani* Viereck (Hymenoptera: Branconidae) were reared on aphids in a population cage on each of the four treatments to avoid learning effects. Parasitoids were reared for five generations before the experiment and fed with *ad libitum* honey-water. In order to obtain uniform age *A. colemani*, mummies were collected from respective lettuce plants and placed individually in gelatin capsules. Upon emergence, female parasitoids were kept for 24 h with male parasitoids to ensure mating, fed *ad libitum* with drops of pure honey, and then used for the experiments. Only female *A. colemani* was used in this experiment. 

*Insects for behavior assays*. The insects used in this experiment were the aphid *M. persicae*, the parasitoid *Aphidius colemani* and the ladybird *Adalia bipunctata* Linnaeus (Coleoptera: Coccinellidae). All insects for this experiment were reared on Brussels sprouts *Brassica oleracea*, with the exception of the aphids for the escape behavior experiment which were reared on the lettuce variety Little Gem. Insects were reared on Brussels sprouts to ensure that they were naive (no maternal influences or learning experience affecting preference behavior). Parasitoids were reared on *M. persicae* as described above. Parasitoids were exposed to the experience of oviposition to enhance responsiveness to the host location cues. All parasitoids tested in the experiment were 48 h old, and experienced solely with *M. persicae* reared on Brussels sprouts. The *A. bipunctata* were purchased from Green Gardener (Yarmouth, UK) and reared in the laboratory for one week before they were used for the experiment. Ladybirds were fed with *M. persicae* and prior to the experiments, they were starved for 12 h.

### 2.2. Effect of Asymptomatic Infection on Plant Traits

Plant height was measured on the first and last day (day 30) of the experiment (N = 30 for each treatment). At the end of the experiment, plant chlorophyll content was measured at three different positions on plant leaves using a handheld chlorophyll meter (Model atLeaf; FT Green LLC) [65,66]. Plants were then harvested and dried in an oven at 75 °C until reaching constant mass (approximately 48 h), and weighed using an electronic balance (Sartorius LC 6200S, Goettingen, Germany). The root: shoot ratio was calculated by dividing the dry mass of individual plant above ground material by the dry mass of the roots.

### 2.3. Effect of Asymptomatic Plant Pathogen Infection on Insects 

*Aphid fecundity, longevity, and size*. Leaf clip-cages (30 mm in diameter by 10 mm in height; [67]) were used to prevent aphid escape. Both clip cage rings were covered with fine muslin netting to allow air to flow to both leaves and aphids. Clip cage edges were lined with foam as a precaution against leaf damage. Adult apterous aphids were randomly chosen from the rearing colonies and one was placed into each individual clip cage, which was attached to a healthy, mature leaf. Aphids were permitted to produce nymphs for 24 h, then the adult and surplus nymphs were removed, leaving five aphid nymphs that were then allowed to grow until they reached maturity. 

To evaluate aphid fecundity, the number of offspring produced by each individual aphid was recorded once every two days and these were removed; this was repeated five times (i.e., 10 day fecundity recorded). To measure aphid longevity and size, the same methods as above were used with ten nymphs, which were allowed to grow to maturity in individual clip cages, taking approximately seven days. Aphids were then collected and transferred into a Petri dish and kept without a food source or water to time of death. Observations were made every 12 h until all aphids died. Aphid hind tibia length was measured using a high-performance stereomicroscope (Leica, MZ9.5, Houston, TX, USA). 

*Parasitoid fecundity, longevity, and size*. Two mated female parasitoids were introduced to forty ten-day-old (4th instar) aphids growing on lettuce plants, and then covered with a mesh plastic bag. Thirty replicates were set up per treatment. Parasitoids were left to oviposit for 24 h before removal. After 10 days, mummies were collected and counted on each of the plants. The proportion of aphids that were mummified was used as the measure of parasitism rates. The mummies were placed individually in a gelatin capsule (16 × 5 mm) and kept in a CE room (described above) until they emerged. Observations were made at 12 h intervals until all of the parasitoids had died. The time taken for parasitoids to emerge and die was recorded. The left hind tibia length of each parasitoid was then measured using a high-performance stereomicroscope (Leica, MZ9.5, Houston, TX, USA). 

### 2.4. Preference Behavior Experiment

*The olfactometer*. The trials were conducted in a four arm olfactometer (BLM4-300, Shanghai Billion Instrument Co. Ltd., Shanghai, China). The internal diameter of the olfactometer was 200 mm and 15 mm deep. The exposure arena was divided into five different zones; one central and four arm zones. Each arm has an inlet to which odors were applied. All four of the olfactometer arms were connected by a silicone tube to a plastic container which contained the odor sources. A vacuum pump was set to exhaust air from the center of the arena at a flow of 250 mL/min per arm. Airtight seals at the inlet of each jar, were used to avoid egress of external odors during the experiments. Before entering the tunnel, air was filtered through a 5-cm thick layer of activated charcoal. Odor-emitting samples were placed in a 3 L plastic container linked by a plastic tube to the relevant olfactometer arm. 

*Preference bioassays*. The preference behavior experiment consisted of exposing aphid, parasitoid, and predators to stimuli derived from simultaneous odor sources: (1) uninfected plant (2) asymptomatic-infected plant and (3) empty arms. The location of the tested plant in the olfactometer was randomly exchanged for each replicate to avoid physical bias. Tests were replicated 30 times for each insect, using different insects and plants in every trial. Twenty aphids, or twenty parasitoids, or one predator were used for each replicate. Plants used for parasitoid and predator preference behavior were infested with 200 adult aphids on each plant to encourage searching behavior. 

The olfactometer was run for five minutes before each trial began to ensure a good circulation of odors. Insect choice was deemed to have been made when the insects fully left the arena and entered one of the collecting jars. This bioassay was carried out in a CE room at 20 ± 1 °C and 60–70% R.H in the dark to eliminate any possible visual cues. The olfactometer arena and its arms were cleaned with 70% alcohol and rinsed with distilled water between each replicate. 

*Escape behavior.* The escape behavior of *M. persicae* fed on uninfected and asymptomatic-infected plants was assessed in a CE room at 20 ± 1 °C and 60–70% R.H. Forty adult aphids were placed on each experimental plant. The lettuce variety Little Gem was used as this plant has a more open growth form, and fewer refugia for test aphids. Aphids were exposed to one foraging *A. bipunctata* or an artificial stimulus as a control. A single ladybird was released at the base of the lettuce plant and allowed to search for aphids for five minutes. If experimental ladybirds failed to forage, the trial was stopped and the replicate was discarded and replaced. For the control treatment, plants were slowly shaken by hand for five seconds to give an artificial stimulus, in an attempt to replicate normal plant movement. Aphids that escaped by dropping off the plant were recorded. Each treatment was replicated 30 times. 

*Confirming asymptomatic infection*. The bioassay to confirm plant health status was made before the plant was harvested. To test for the presence of systemic infection by *B. cinerea*, three mature leaf samples from each plant (1 cm in diameter) with no visible symptoms of infection were randomly harvested at the end of the experiment from each experimental plant. Leaf samples were first disinfected with 70% ethanol for one minute, and then in a 20% solution of bleach (Domestos, Unilever: 5% NaOCl in alkaline solution with surfactants) for one minute. Samples were then rinsed three times in sterile distilled water and allowed to dry. This removes all surface inoculum, whether dusted or soaked in [68]. The leaf disk was then plated on a *Botrytis* Selective Media (BSM) agar and incubated at 18–20 °C for at least 10 days in an incubator with alternating UV-A light (12 h/day) and dark (12 h/day) to determine the presence or absence of *B. cinerea*. Confirmation of presence was based on the sporulation of the pathogen and morphological observation of fungal colonies under a high-performance stereomicroscope (Leica MZ9.5, Houston, TX, USA). 

### 2.5. Statistical Analyses

All statistical analyses were conducted using R-statistical software version 3.4.0 [69]. The influence of plant variety and pathogen infection on plant traits, and aphid/parasitoid size and longevity were compared using Linear Models (LM), while aphid total fecundity was analyzed using Generalized Linear Models (GLM) with Poisson errors. The proportion of parasitized aphids was analyzed by using GLM with quasibinomial errors. The significance of differences between mean values were determined by using LSmeans and separation by post-hoc Tukey tests, with plant variety and infection status as explanatory variables. 

*Behavior*. The preference behavior of the aphids, predatory ladybirds, and parasitoid wasps towards the experimental target were calculated. The preference of the insects (measured as proportion choosing a given arm for aphids and parasitoids, while attraction of individual ladybirds was modelled as a binary response count) towards plant and blank odor was analyzed using a generalized linear model (glm) with quasibinomial error; and either insect choosing infected, uninfected or blank odor were analyzed using multinomial logistic regression analysis. The escape behavior of aphids was analyzed using a generalized linear model (glm) with a quasibinomial error structure. 

## 3. Results

### 3.1. Life History Effects

*Plants*. Plant varieties differed in their physical traits (Table 1; N = 30 for each treatment), with Tom Thumb showing a lower chlorophyll index, plant dry weight and plant height than Little Gem (Table 2). Asymptomatic infection by *Botrytis cinerea* resulted in reduced chlorophyll and plant dry weight for both plant varieties, but there was no effect on plant height (Table 2). There was no effect of plant variety or of infection status on root:shoot ratios. All interaction terms were non-significant.

**Figure 1:**
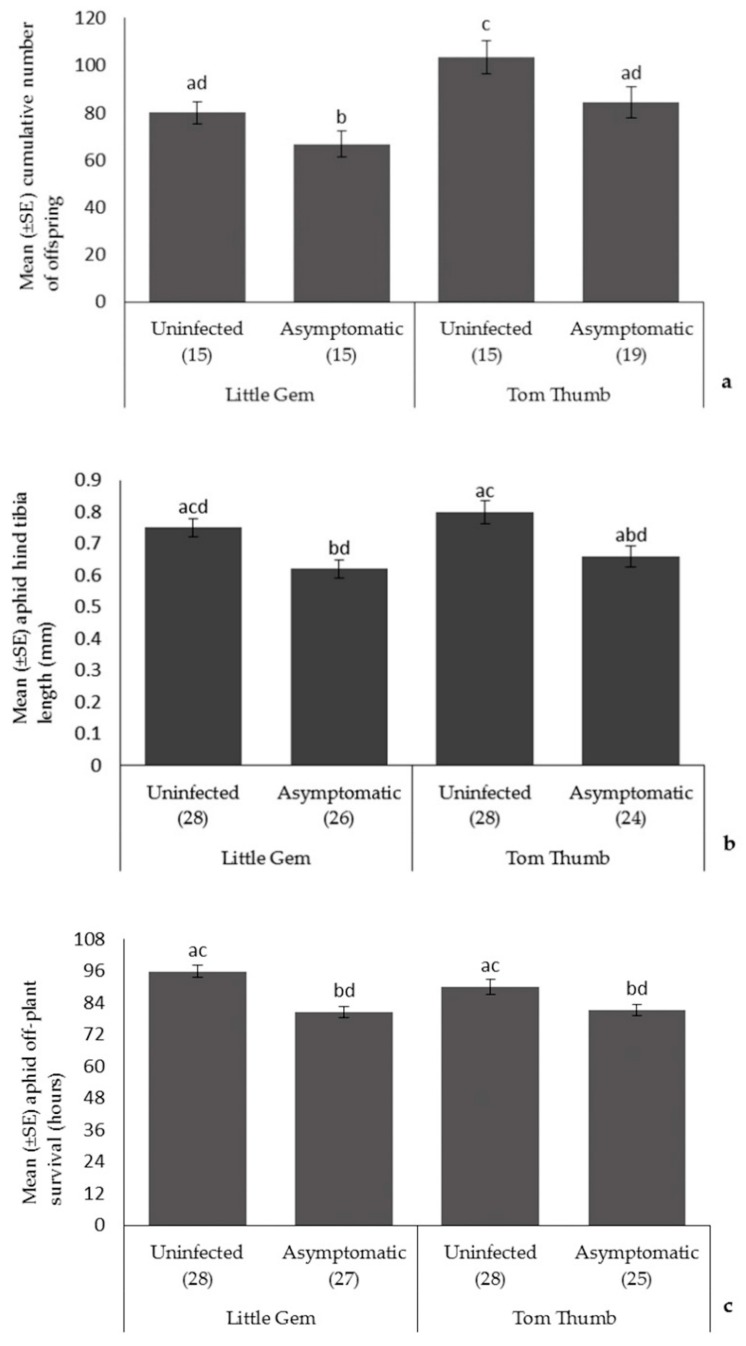
The effect of asymptomatic *B. cinerea* infection status and plant variety on Mean ± SE (**a**) cumulative number of aphid offspring; (**b**) aphid hind tibia length; and (**c**) aphid off-plant survival. Number of replicates per treatment is shown below each bar; treatments sharing the same letters above each bar are not significantly different at *p* < 0.05 following post-hoc tests.

*Aphids*. Plant variety influenced the number of aphids produced, with aphid fecundity higher on Tom Thumb. Aphid size and off-plant survival time did not differ with plant variety (Table 3; Figure 1). Asymptomatic plant pathogen infection significantly reduced aphid fecundity, size, and off-plant survival time. Overall, aphids had the best performance when reared on uninfected Tom Thumb plants, and the poorest when reared on infected Little Gem. All interaction terms were non-significant. 

**Figure 2:**
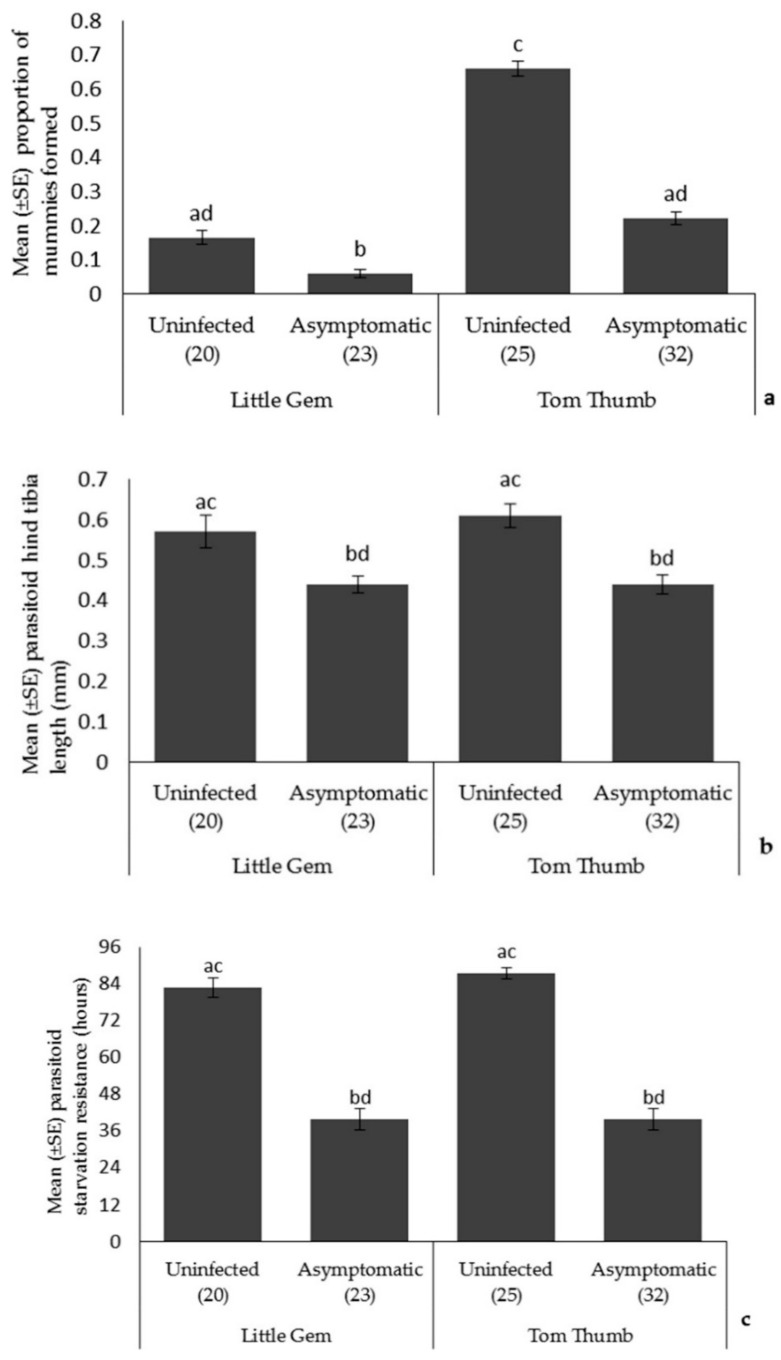
The effect of asymptomatic *B. cinerea* infection status and plant variety on Mean ± SE (**a**) proportion of parasitoid mummies formed; (**b**) parasitoid hind tibia length; and (**c**) parasitoid starvation resistance. Number of plant replicates per treatment is shown below each bar; treatments sharing the same letters above each bar are not significantly different at *p* < 0.05 following post-hoc tests.

*Parasitoids*. Plant variety influenced parasitoid attack rates (more mummies on Tom Thumb), but there was no effect of plant variety on parasitoid size or longevity (Table 4; Figure 2). The proportion of parasitoid mummies formed on asymptomatically infected plants was lower than that found on uninfected plants, and there was also a significant interaction between plant variety and infection status on parasitoid attack rates. Parasitoids emerging from aphids reared on asymptomatically infected plants were smaller and showed reduced starvation resistance. 

### 3.2. Behavior

**Figure 3:**
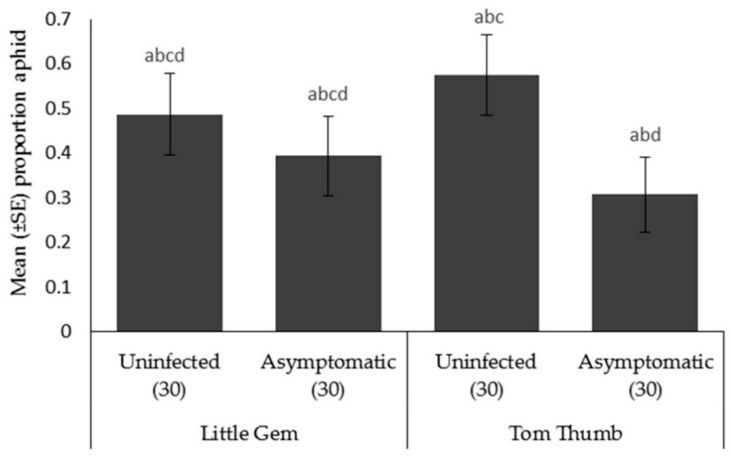
Mean ± SE proportion of aphids *Myzus persicae* orientating in an olfactometer trial towards two varieties (Little Gem, Tom Thumb) of uninfected or asymptomatically infected lettuce plants. Number of replicates per treatment is shown below each bar; treatments sharing the same letters above each bar are not significantly different at *p* < 0.05 following post-hoc tests.

*Aphid choice.* Aphids preferred to move towards plant odors compared to the blank arm. When choosing between different host plant possibilities, aphids were significantly more likely to choose uninfected Tom Thumb than the asymptomatically infected Tom Thumb. However, aphids showed no preference between uninfected and asymptomatically infected Little Gem (Table 5; Figure 3). 

**Figure 4:**
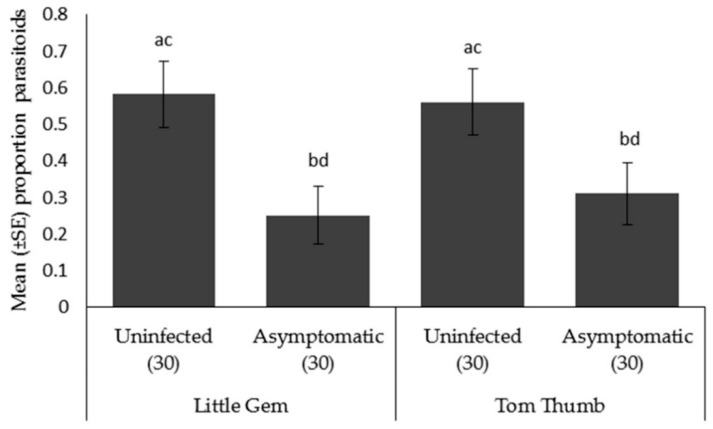
Mean ± SE proportion of parasitoids *Aphidius colemani* orientating in an olfactometer trial towards aphids on two varieties (Little Gem, Tom Thumb) of uninfected or asymptomatically infected lettuce plants. Number of replicates per treatment is shown below each bar; treatments sharing the same letters above each bar are not significantly different at *p* < 0.05 following post-hoc tests.

*Parasitoid choice*. *Aphidius colemani* showed a preference towards aphid/plant odor sources as opposed to a blank odor sources for both Tom Thumb and Little Gem. When given a preference between plants, parasitoids significantly preferred aphids on uninfected Tom Thumb and uninfected Little Gem compared to the corresponding asymptomatically infected plants (Table 6; Figure 4).

*Predator choice*. Significantly more ladybirds oriented towards the Tom Thumb odor source (mean/SE: 0.733 ± 0.082) as opposed to a blank odor source (mean/SE: 0.266 ± 0.082), and to Little Gem (mean/SE: 0.633 ± 0.089) as opposed to a blank odor (mean/SE: 0.366 ± 0.089). The presence of asymptomatic pathogen infection on both lettuce varieties also did not influence the preference behavior of *A. bipunctata* [Uninfected Tom Thumb (mean/SE: 0.433 ± 0.092), Infected Tom Thumb (mean/SE: 0.300 ± 0.085), Blank (mean/SE: 0.266 ± 0.082); Uninfected Little Gem (mean/SE: 0.366 ± 0.089), Infected Little Gem (mean/SE: 0.266 ± 0.082), Blank (mean/SE: 0.366 ± 0.089] (Table 7).

**Figure 5:**
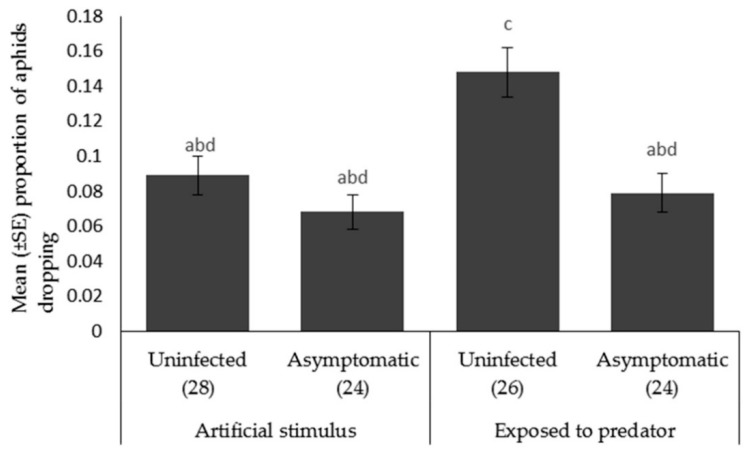
Mean ± SE proportion of *Myzus persicae* showing escape behavior (dropping) in response to an artificial stimulus (gentle shaking) and the presence of a foraging ladybird, *Adalia bipunctata* on uninfected or asymptomatically infected lettuce plants (var. Little Gem). Number of replicates per treatment is shown below each bar; treatments sharing the same letters above each bar are not significantly different at *p* < 0.05 following post-hoc tests.

*Aphid escape behavior.* The proportion of aphids that dropped when *A. bipunctata* was present (F_1,99_ = 9.229, *p* < 0.003) was significantly higher than the proportion dropped when the plant was shaken. There was a significant effect of plant pathogen infection on the proportion of aphids falling from the plant (F_1,100_ = 13.524, *p* < 0.001) (Figure 5), with the aphids fed on the uninfected plants dropping more frequently than those fed on asymptomatically infected plants.

## 4. Discussion

Asymptomatic infection by plant pathogens is likely to be widespread, yet we have almost no understanding of its effects on species interactions at higher trophic levels. Here, we show that asymptomatic host plant infection by *B. cinerea* of two lettuce varieties affects species at three trophic levels. While our lettuce plants showed no visible symptoms of infection, it is apparent that this hidden, asymptomatic infection did affect host plants. Infected, asymptomatic plants had reduced chlorophyll content and showed reduced mass. Therefore, while effects were minimal and symptoms of disease absent, it is evident that asymptomatic infection did affect host plants, and this was consistent across host plant varieties. However, the consequences of asymptomatic infection on our model insect herbivore was clear. Aphids reared on infected asymptomatic plants were smaller, produced fewer offspring and had reduced off-plant survival times. In turn, parasitoids reared on hosts feeding on infected asymptomatic plants, showed reduced attack rates, and their offspring were smaller and also showed reduced longevity. While there were differences in traits between the lettuce varieties, this only appeared to affect aphid fecundity and parasitoid attack rate. Surprisingly, these differences were not fully reflected in the choices made by the aphid *Myzus persicae* and two of its enemies, the ladybird *Adalia bipunctata* and the parasitoid *Aphidius colemani*, a species widely used as a biocontrol agent. Aphids preferentially chose uninfected Tom Thumb plants over asymptomatically infected plants, but did not distinguish between infected and uninfected Little Gem plants. Ladybirds did choose both Little Gem and Tom Thumb aphids/plants over empty controls, but did not distinguish between infected and uninfected plants/aphids. In contrast, parasitoids did prefer plants with aphids over controls, and furthermore showed a strong preference for uninfected plants and their aphids over asymptomatically infected plants and aphids irrespective of plant variety. Finally, we evaluated aphid escape behavior and found that aphids were less likely to attempt escape from foraging ladybirds when reared on asymptomatically infected plants. 

What is not definitively understood is the underlying causes of these changes. While it is most reasonable to consider that this is a result of changes in plant defenses due to the presence of asymptomatic *B. cinerea*, it is possible, albeit unlikely, that this effect is simply a result of reduced seed quality resulting in poorer quality host plants. Irrespective of the causal factor, what is clear is that being reared on plants asymptomatically infected by *B. cinerea* changes the fitness, behavior and interactions of species at higher trophic levels. The value of this work lies in the demonstration of the importance of hidden disease on insect life history and behavior. Diseased plants, even when asymptomatic, alter the physiology and behavior of insect herbivores and their natural enemies and hence may affect how assemblages are formed and so affect the outcome of biological control efforts. Infection by this pathogen in apparently healthy wild growing host plants such as *T. vulgare* may reach 50% of plant samples [60]. These findings suggest plant pathogens have a strong influence on arthropod tritrophic systems, and are therefore of particular relevance to arthropod biocontrol. 

The effect of *Botrytis* infection on postharvest products [70,71,72,73] and plants under cultivation [55,74,75,76] is well studied. However, little has been reported on the effect of asymptomatic infection on plant traits. An investigation of the effect of *B. cinerea* infection on lettuce plants reported that latent *B. cinerea* infection on seed, root, stem, and leaf was common [56]. Asymptomatic lettuce with more *Botrytis* recovery were of greater mass than uninfected plants [60]. This is inconsistent with the findings here, but the methods differ in the two studies. 

Aphid size, off-plant survival time and fecundity were reduced when reared on asymptomatic, infected plants (Table 3). If the presence of the pathogen either directly or indirectly results in a reduction in host plant quality, then such effects are not unexpected. Due to the reduction in plant quality caused by pathogen infection, it has been suggested that this could play a role in determining the structure of arthropod communities [1,25]. The low quality of diseased plants generally results in a decline in fecundity and an increase in developmental time of insect herbivores [77]. This change results from a reduction in available plant amino acids due to assimilation of resources by *B. cinerea* [49] and possibly from interlinked defense pathways [11]; these effects influence the ability of aphids to effectively utilize the host plant [78,79]. We show that it is not only plants showing symptoms that affect their herbivores’ life histories [80,81,82] but also that asymptomatic host plant infection also alters the growth, reproduction, and starvation resistance of an insect herbivore. 

Interactions between two trophic levels are predicted to have effects on the third trophic level [83,84,85]. In this study system, the effect of pathogen infection on plants may provide a significant biotic factor that indirectly modulates the outcome of interspecific interactions at higher trophic levels. We observed that asymptomatic infection by *B. cinerea* has subsequent effects on our model herbivore, and so we may expect consequent changes at higher trophic levels. Indeed, we demonstrate that the parasitoid *A. colemani* was negatively affected by asymptomatic infection, exhibiting a reduction in parasitism rate, growth rate, and starvation resistance, suggesting that the consequences of such hidden infections may ramify through trophic interactions, although we do not know if this is a simple consequence of host size reduction, or some more subtle change in plant/pathogen chemistry. Nevertheless, asymptomatic plant pathogen infection may alter patterns of plant-herbivore and host-parasitoid interactions in natural and agro-ecosystems, with implications for biological control programs. 

*Botrytis* infected plants can produce symptoms of infection such as a fast-spreading soft rot, which under favorable conditions can completely destroy plant tissues in less than 72 h [44]. As plants produce specific volatiles as a response to pathogen infection [86,87], this will provide host recognition cues for parasitoids [88,89]. Parasitoid host preference was correlated with host suitability for offspring development [90,91], where parasitoid females maximize their fitness by locating the best insect host and/or their habitat to ensure the successful development of their progeny. 

Contrary to the preference behavior shown by the aphid *M. persicae* and its parasitoid *A. colemani*, our predatory insect *A. bipunctata* was not affected by the presence of asymptomatic *Botrytis* infection, and furthermore, for the Little Gem variety, showed no difference in preference between arms with plants and the empty controls. The latter observation again suggests that Little Gem produces fewer volatiles, even when attacked by aphids. Generally, aphid predators depend on the chemical cues emitted by their potential prey and the plant associated with their prey, alone or in association [92,93,94]. In contrast to parasitoids, where developing offspring may be lost if the plant succumbs to disease before emergence, adult ladybirds can directly benefit from consuming prey, and their mobile offspring may be able to leave the plant before the host plant perishes. 

Asymptomatic infection by *Botrytis* may not affect *A. bipunctata* host choice, but it does affect *M. persicae* escape behavior from these predators. Antipredator behavior, such as dropping, kicking or walking away from predators, are fitness-related [95]. Given that aphids reared on asymptomatically infected plants showed reduced off-plant survival times, it is not surprising that dropping behavior (a trade-off between predation risk and of finding a suitable host plant before death through starvation or predation; [96]) was reduced. Aphid dropping is therefore a risky and energetically costly antipredator behavior [97] and when the energetic stress of aphids is increased, aphid antipredator responses change from walking away and dropping to kicking behavior [98]. Similar to our findings, the aphids *Acyrthosiphon pisum* and *Uroleucon jaceae* reduce their dropping rate when feeding on low-quality plants [99].

Variation in plant quality resulting from differences in plant genotype plays an important role in shaping arthropod community structure [100,101]. Such effects may be mediated by either the nutritional, defensive, or physical qualities of the host plant. What is of interest here is whether there is an interaction between host plant variety and infection status. We found that the performance of both the aphids and their parasitoids differed between plant varieties, with the performance being better on lettuce variety Tom Thumb than on Little Gem. In part the latter may be the result of the differing growth forms (Little Gem is a relatively tight-headed Cos lettuce, while Tom Thumb is more open in structure) affecting parasitoid foraging behavior. While this explains differences between the varieties, the effect of asymptomatic infection remains. In addition, we used one clone of *Myzus persicae* in this study. It is worth considering the interaction between variation among aphid clones in traits such as resistance to parasitoid attack, escape behavior, or competitive ability, and the presence of hidden pathogen infection, as these affect the ecological interactions of aphids in the field [102,103,104]. Unpicking the effects of such factors will prove worthwhile if we are to better understand the potential effects of asymptomatic infection on insect pest management.

## 5. Conclusions

Whether because they are assumed, ignored, or dismissed, the ecological consequences of plant-pathogen-insect interactions and their importance is poorly understood. Here, we present experimental evidence that demonstrates that asymptomatic infection by a widespread, economically important plant pathogen can play an important role in determining the interaction between insect herbivores and their natural enemies. A very wide diversity of plant species host infections by *B. cinerea*, which may cause no visible symptoms on the plant at the initial time of infection [60]. This study suggests that hidden plant infections may have considerable direct and indirect effects on the structuring of species assemblages in both natural and agro-ecosystems. A challenge for the future is to consider how such effects may scale up to the larger processes that help determine insect population dynamics, particularly in the context of biological control. Latent and asymptomatic infection by plant pathogens are likely to be widespread in nature; this is an early step in developing an understanding of the consequences of such hidden infections in the field.

## Figures and Tables

**Table 1:** Summary of effects of asymptomatic *B. cinerea* infection status and plant variety on plant traits following analysis. Significant values are in bold.

Plant Trait	d.f.	Explanatory Variable	Coefficient z Value ± SE	*p*
atLEAF value		**Intercept**	**38.747 ± 0.648**	**<0.001**
	1	**Variety**	**−6.427 ± 0.935**	**<0.001**
	1	**Infection status**	**7.713 ± 0.909**	**<0.001**
	1	Interaction	−0.215 ± 1.298	0.830
Shoot:root		**Intercept**	**20.786 ± 0.027**	**0.001**
	1	Variety	1.787 ± 0.032	0.088
	1	Infection status	0.984 ± 0.044	0.327
	1	Interaction	−0.175 ± 0.064	0.861
Dry weight (g)		**Intercept**	**21.022 ± 0.558**	**<0.001**
	1	**Variety**	**−4.442 ± 0.805**	**<0.001**
	1	**Infection status**	**5.744 ± 0.783**	**<0.001**
	1	Interaction	−1.280 ± 1.118	0.204
Plant height (mm)		**Intercept**	**98.515 ± 1.606**	**<0.001**
	1	**Variety**	**−34.003 ± 2.316**	**<0.001**
	1	Infection status	−0.875 ± 2.251	0.384
	1	Interaction	0.948 ± 3.216	0.345

**Table 2:** The effect of asymptomatic *B. cinerea* infection and plant variety on plant traits. atLEAF value represents the amount of chlorophyll present in the plant leaf. For each parameter, differences among treatment were examined by post-hoc Tukey tests (*p* < 0.05). Means within columns followed by the same letters are not significantly different.

Treatment	Plant Traits (Mean ± SE)			
	atLEAF Value	Shoot:Root	Dry Weight (g)	Plant Height (mm)
Uninfected Little Gem	32.15 ± 0.70 ^a^	0.62 ± 0.02 ^a^	16.25 ± 0.74 ^a^	156.25 ± 2.00 ^a^
Asymptomatic Little Gem	25.14 ± 0.62 ^b^	0.57 ± 0.02 ^a^	11.75 ± 0.50 ^b^	158.22 ± 1.27 ^a^
Uninfected Tom Thumb	25.86 ± 0.70 ^b^	0.68 ± 0.03 ^a^	11.23 ± 0.42 ^b^	80.54 ± 1.50 ^b^
Asymptomatic Tom Thumb	19.14 ± 0.52 ^c^	0.65 ± 0.05 ^a^	8.48 ± 0.50 ^c^	79.46 ± 1.53 ^b^

**Table 3:** Summary of effects of asymptomatic *B. cinerea* infection status and plant variety on aphid traits following analysis. Significant values are in bold.

Aphid Trait	d.f.	Explanatory Variable	Coefficient Value ± SE	*p*
Cumulative number of offspring		**Intercept**	**132.904 ± 0.031**	**<0.001**
	1	**Host plant variety**	**5.821 ± 0.040**	**<0.001**
	1	**Host plant infection status**	**4.236 ± 0.042**	**<0.001**
	1	Interaction	0.408 ± 0.055	0.683
Hind tibia length		**Intercept**	**18.907 ± 0.032**	**<0.001**
	1	Host plant variety	0.894 ± 0.042	0.373
	1	**Host plant infection status**	**2.862 ± 0.130**	**0.005**
	1	Interaction	0.110 ± 0.007	0.912
Off-plant survival time		**Intercept**	**34.454 ± 2.336**	**<0.001**
	1	Host plant variety	0.221 ± 3.369	0.826
	1	**Host plant infection status**	**4.668 ± 3.274**	**<0.001**
	1	Interaction	−1.393 ± 4.677	0.167

**Table 4:** Summary of effects of asymptomatic *B. cinerea* infection status and plant variety on parasitoid traits following analysis. Significant values are in bold.

Parasitoid Traits	d.f.	Explanatory Variable	Coefficient Value ± SE	*p*
Proportion of mummies formed		**Intercept**	**−12.415 ± 0.221**	**<0.001**
	**1**	**Aphid host plant variety**	**6.107 ± 0.246**	**<0.001**
	**1**	**Aphid host plant infection status**	**4.215 ± 0.269**	**<0.001**
	**1**	**Interaction**	**2.527 ± 0.308**	**0.013**
Hind tibia length		**Intercept**	**14.447 ± 0.031**	**<0.001**
	1	Aphid host plant variety	−0.019 ± 0.040	0.985
	**1**	**Aphid host plant infection status**	**2.828 ± 0.045**	**0.005**
	1	Interaction	0.563 ± 0.060	0.574
Starvation resistance		**Intercept**	**12.845 ± 3.818**	**<0.001**
	1	Aphid host plant variety	−1.857 ± 5.006	0.066
	**1**	**Aphid host plant infection status**	**6.029 ± 5.599**	**<0.001**
	1	Interaction	1.864 ± 7.432	0.065

**Table 5:** Summary of effects of plant infection status on aphid host plant preference behavior on two lettuce varieties. N = 30 for each treatment. Significant values are in bold.

Plant Variety	d.f.	Choice	Coefficient t Value ± SE	*p*
Tom Thumb	**1**	**Plant vs. Blank**	**8.394 ± 0.246**	**<0.001**
	**1**	**Infected plant vs. Blank**	**−0.926 ± 0.191**	**<0.001**
	**1**	**Uninfected plant vs. Blank**	**−1.549 ± 0.178**	**<0.001**
	**1**	**Infected plant vs. Uninfected plant**	**−0.623 ± 0.126**	**<0.001**
Little Gem	**1**	**Plant vs. Blank**	**8.588 ± 0.223**	**<0.001**
	**1**	**Infected plant vs. Blank**	**1.190 ± 0.205**	**<0.001**
	**1**	**Uninfected plant vs. Blank**	**1.402 ± 0.200**	**<0.001**
	1	Infected plant vs. Uninfected plant	0.211 ± 0.133	0.112

**Table 6:** Summary of effects of asymptomatic *B. cinerea* infection status and plant variety on parasitoid host preference behavior with aphids reared on two lettuce varieties. N = 30 for each treatment. Significant values are in bold.

Plant Variety	d.f.	Response Variable	Coefficient t Value ± SE	*p*
Tom Thumb	**1**	**Plant vs. Blank**	**8.621 ± 0.222**	**<0.001**
	**1**	**Infected plant vs. Blank**	**0.882 ± 0.198**	**<0.001**
	**1**	**Uninfected plant vs. Blank**	**−1.472 ± 0.184**	**<0.001**
	**1**	**Infected vs. Uninfected plant**	**−0.590 ± 0.133**	**<0.001**
Little Gem	**1**	**Plant vs. Blank**	**8.74 ± 0.185**	**<0.001**
	**1**	**Infected plant vs. Blank**	**0.420 ± 0.194**	**<0.030**
	**1**	**Uninfected plant vs. Blank**	**1.259 ± 0.170**	**<0.001**
	**1**	**Infected vs. Uninfected plant**	**−0.838 ± 0.146**	**<0.001**

**Table 7:** Summary of effects of asymptomatic *B. cinerea* infection status on predator preference behavior when offered prey reared on two lettuce varieties. N = 30 for each treatment. Significant values are in bold.

Plant Variety	d.f.	Response Variable	Coefficient z Value ± SE	*p*
Tom Thumb	**1**	**Plant vs. Blank**	**3.465 ± 0.583**	**<0.001**
	1	Infected plant vs. Blank	0.286 ± 0.573	<0.774
	1	Uninfected plant vs. Blank	1.343 ± 0.553	<0.179
	1	Infected vs. Uninfected plant	−1.067 ± 0.542	<0.286
Little Gem	**1**	**Plant vs. Blank**	**2.040 ± 0.535**	**<0.041**
	1	Infected plant vs. Blank	−0.830 ± 0.560	<0.407
	1	Uninfected plant vs. Blank	−0.001 ± 0.533	<0.999
	1	Infected vs. Uninfected plant	−0.830 ± 0.560	<0.407
